# Reef Sharks Exhibit Site-Fidelity and Higher Relative Abundance in Marine Reserves on the Mesoamerican Barrier Reef

**DOI:** 10.1371/journal.pone.0032983

**Published:** 2012-03-08

**Authors:** Mark E. Bond, Elizabeth A. Babcock, Ellen K. Pikitch, Debra L. Abercrombie, Norlan F. Lamb, Demian D. Chapman

**Affiliations:** 1 School of Marine and Atmospheric Sciences, SUNY Stony Brook, Stony Brook, New York, United States of America; 2 Institute for Ocean Conservation Science, School of Marine and Atmospheric Sciences, SUNY Stony Brook, Stony Brook, New York, United States of America; 3 Rosenstiel School of Marine and Atmospheric Sciences, University of Miami, Miami, Florida, United States of America; 4 Abercrombie and Fish, Port Jefferson Station, New York, United States of America; 5 Absolondna, Dangriga, Belize; Ecole Normale Supérieure de Lyon, France

## Abstract

Carcharhinid sharks can make up a large fraction of the top predators inhabiting tropical marine ecosystems and have declined in many regions due to intense fishing pressure. There is some support for the hypothesis that carcharhinid species that complete their life-cycle within coral reef ecosystems, hereafter referred to as “reef sharks”, are more abundant inside no-take marine reserves due to a reduction in fishing pressure (i.e., they benefit from marine reserves). Key predictions of this hypothesis are that (a) individual reef sharks exhibit high site-fidelity to these protected areas and (b) their relative abundance will generally be higher in these areas compared to fished reefs. To test this hypothesis for the first time in Caribbean coral reef ecosystems we combined acoustic monitoring and baited remote underwater video (BRUV) surveys to measure reef shark site-fidelity and relative abundance, respectively. We focused on the Caribbean reef shark (*Carcharhinus perezi*), the most common reef shark in the Western Atlantic, at Glover's Reef Marine Reserve (GRMR), Belize. Acoustically tagged sharks (N = 34) were detected throughout the year at this location and exhibited strong site-fidelity. Shark presence or absence on 200 BRUVs deployed at GRMR and three other sites (another reserve site and two fished reefs) showed that the factor “marine reserve” had a significant positive effect on reef shark presence. We rejected environmental factors or site-environment interactions as predominant drivers of this pattern. These results are consistent with the hypothesis that marine reserves can benefit reef shark populations and we suggest new hypotheses to determine the underlying mechanism(s) involved: reduced fishing mortality or enhanced prey availability.

## Introduction

Many tropical nations are turning to marine reserves to help maintain coral reef biodiversity, ecosystem function, ecotourism and fisheries [1]–[4]. Marine reserves can clearly enhance exploited coral reef species that have relatively sedentary adult life-stages, in which some individuals live almost exclusively within reserve boundaries (i.e., reef-associated bony fish and invertebrates [2], [5]–[8]). This enhancement occurs because the reserve provides a respite from fishing mortality that leads to an increase in local abundance and reproductive output [2], [5]–[8]. However, can marine reserves also benefit large, roving reef predators that are potentially mobile throughout their life? This group includes sharks, which make up a significant fraction of the top predators on relatively pristine coral reefs [9], [10]. Sharks are currently experiencing intense fishing pressure worldwide, largely due to the Asian shark fin trade [11], which is worrisome in light of their relatively low reproductive potential [12], [13].

There is a modest body of data supporting the hypothesis that marine reserves can benefit certain shark populations [14]–[19]. Most of the focal species of these prior studies belong to the family Carcharhinidae (requiem or whaler sharks) and complete their life-cycle within coral reef ecosystems. Species with these general characteristics are hereafter referred to as “reef sharks”. No temporal monitoring studies have been conducted to show an increase in reef shark abundance following marine reserve establishment. However, existing studies can be divided into those demonstrating that reef sharks reside inside reserves and those showing differences in reef shark relative abundance between reserves and fished sites. Juvenile Caribbean reef sharks (*Carcharhinus perezi*) in Brazil were more abundant inside than immediately outside a marine reserve at an oceanic archipelago [14]. Acoustic monitoring of several individuals revealed year round residency to small home ranges within the reserve, indicating that this protected area reduced the exposure of these individuals to fisheries [15]. On Australia's Great Barrier Reef surveys of reef sharks (mainly grey reef, *C. amblyrhynchos* and whitetip reef, *Triaenodon obesus*) revealed higher relative abundance of sharks inside than outside parts of the reef that are zoned for no entry or no fishing [16]–[18]. Limited acoustic monitoring, however, suggests that long range movements between reefs and across marine reserve/fishing zones may be common at least among large juveniles and adults in some of these species [19], which raises some questions about how marine reserves are contributing to the observed spatial abundance pattern. It is possible that juvenile site-fidelity is high enough to drive the observations of increased shark abundance in these areas, even though large juveniles and adults are vulnerable to fishing as they move between management zones [19].

Marine reserves are increasingly being used for marine conservation in the Caribbean [6], [7], yet very little is known about the effectiveness of this strategy in conserving the regional shark fauna. A recent survey of recreational SCUBA divers in the Caribbean found that shark sightings are quite rare, except for some places that have shark conservation regulations or large marine reserves in place [20]. Relatively few shark sightings occurred in the Mesoamerican Barrier Reef area of this survey [20] even though this region has a relatively large number of marine reserves. For example, Glover's Reef atoll is a large, zoned marine reserve that has been protected since 1996 [6]. A stable catch-per-unit effort (CPUE) of Caribbean reef sharks was reported on research longlines set in Glover's Reef Marine Reserve (GRMR) from 2001–2005, which suggests that reserve protection may be maintaining reef sharks in this location [21]. Short term (150 day) acoustic monitoring of 4 individuals (2 adults, 2 juveniles) of this species showed they were residential to GRMR and could benefit from reserve protection over at least this time-scale [22]. However, one adult male made a short term (4 day) return movement between GRMR and a nearby fished atoll across 30 km of open water [22], while several other adult individuals moved into deep water off the reef platform outside of the reserve boundary [23]. These telemetry studies suggest that movements outside of reserve boundaries might undermine reserve protection for this species, as has been suggested for congeners in the Indo-Pacific [19]. More information on shark movements and relative abundance in different management zones is needed to understand the extent to which marine reserves benefit Caribbean reef sharks and reef sharks in general.

Here we combined acoustic monitoring with baited remote underwater video (BRUV) to examine site-fidelity and relative abundance of Caribbean reef sharks in a marine reserve in Belize (GRMR). Given the hypothesis that Caribbean reef shark populations can benefit from no-take marine reserves and increase in abundance in these areas, we predicted that (1) acoustically tagged Caribbean reef sharks at GRMR would exhibit site-fidelity to the reserve and (2) the relative abundance of Caribbean reef sharks would be higher at GRMR (and other reserve reefs) when compared to fished reefs.

## Materials and Methods

### Study species

The Caribbean reef shark (*Carcharhinus perezi*) is a large requiem shark (growing to 295 cm total length) that is endemic to the Western Atlantic from Bermuda to southern Brazil [24], [25]. It is the only carcharhinid in this region that completes its entire life cycle within coral reef ecosystems and is rarely found away from this type of habitat [24], [25]. Caribbean reef sharks do not have geographically discrete nursery areas, instead all life-stages occur over the fore-reef, at depths of 10–30 m [21], [22], [24]–[26]. Adults also frequently occur over the reef slope at depths of at least 352 m, especially during daylight hours [23]. Caribbean reef sharks frequent lagoons associated with coral reefs, but are not typically found in shallow seagrass or mangrove habitats within these lagoons [21], [25]. This species feeds on a wide variety of reef fish and is exploited by humans for the seafood trade [25]. They are also one of the most common sharks observed by SCUBA divers, either naturally or under baited conditions, and are therefore important for the ecotourism industry in many countries [25], [26]. They are considered “Near Threatened” by the International Union for the Conservation of Nature, with a range-wide population trend listed as “Decreasing” [25]. The IUCN assessors of the species indicate that it may meet the criteria for the more serious listing of “Vulnerable” as more fisheries and population trend data become available [25].

### Primary study site

**Figure 1:**
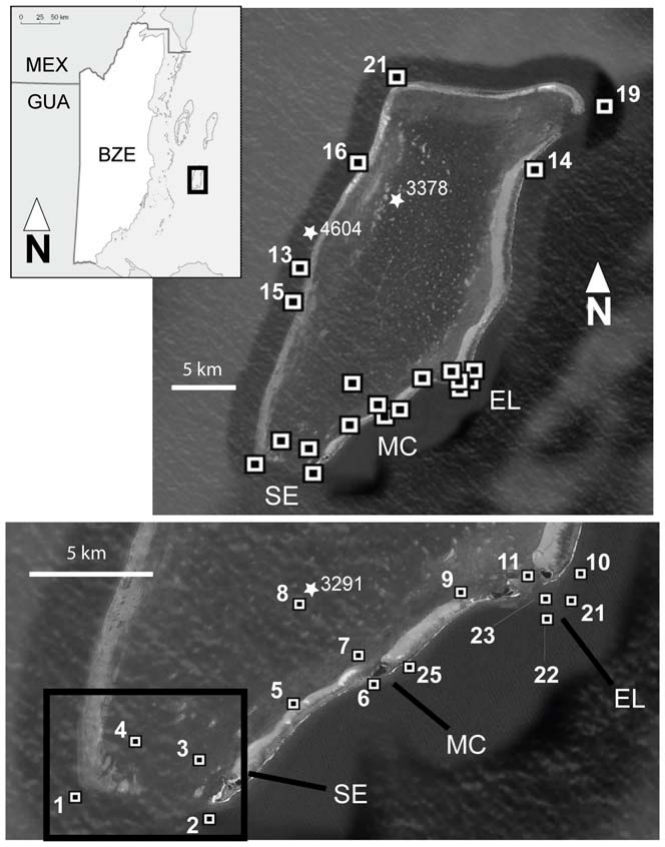
Top left-Belize (BZE) and surrounding nations (MEX = Mexico, GUA = Guatemala). Box contains Glover's Reef Marine Reserve (GRMR). Top right: GRMR showing the location of all receivers (black and white squares). The three primary locations where sharks were tagged are labeled “EL” = Elbow, “MC” = Middle Caye and “SE” = Southern Entrance (see Table 1). Tagging locations for other sharks are denoted by their stars and their tag identification number (see Table 1 for capture and biological information). Bottom: Southern part of the atoll showing more detail of receiver locations.

Glover's Reef Marine Reserve (GRMR) encompasses Glover's Reef Atoll (16°44′N, 87°48′W), which lies approximately 25 km to the east of the Mesoamerican Barrier Reef and 45 km east of mainland Belize (Figure 1). The atoll is approximately 30 km long and at the maximum 10 km wide. The atoll's western reef crest lies submerged with the eastern reef crest being exposed and broken to produce five cuts, which allow shark movements between the ocean reef and lagoon ecosystems. The atoll also includes six sparsely populated cayes. The GRMR was established in 1997 and is comprised of a “no-take zone” on the interior, surrounded by a “general use zone” (32, 834 ha) which prohibits the use of gill-nets and longlines throughout the entire atoll out to the 180 m depth contour [6]. This gear restriction essentially precludes a commercial shark fishery within GRMR, even though hook and line fishing is permitted in the general use zone. Reserve regulations are actively enforced by resident members of a permanent Government of Belize Department of Fisheries station located on Middle Caye.

### Acoustic monitoring at GRMR

Caribbean reef sharks were collected using longlines and fitted with transmitters under permit from the Belize Department of Fisheries (see [21] and [22] for description of capture and handling methods). All animal handling procedures were reviewed and approved by the Belize Ministry of Agriculture and Fisheries (Department of Fisheries) under a series of annual research permits issued from 2000 to the present (most recent #00005-11). We hereafter use total length (TL), the length from the tip of the snout to the tip of the tail as the standard measurement. Individually coded transmitters (V9 for small sharks <110 cm TL, V16 for larger sharks; Vemco Ltd. Nova Scotia, Canada) that had previously been coated in beeswax to alleviate physical irritation and prevent an immunological reaction were implanted into the shark's coelom. All transmitters emitted acoustic pulse trains with a semi-randomized signal delay for between 180–360 seconds. The individual was positioned upside-down until it entered a state of tonic immobility. The transmitter was then inserted through a ∼5 cm incision made just anterior to the origin of one of the pelvic fins. Following implantation, the opening was closed with braided-nylon sutures. Upon completion of surgery the shark was rolled back over, the hook was entirely removed and the shark was released. Circle hooks were used to reduce instances of gut-hooking and the lines were checked frequently (every 90–180 minutes, depending on location) to minimize the physiological stress of capture. In May and October 2006 two adult female sharks (3291, 3292) were each fitted with an external V16 coded transmitter. The transmitter was anchored with a plastic umbrella dart in the shark's dorsal musculature just below the dorsal fin (Pflegler Institute for Environmental Research, CA, U.S.A.) and tethered with a 5 cm length of coated stainless steel wire. Externally mounted transmitters were fitted to these individuals instead of performing intracoelomic insertion because of inclement weather and rough sea conditions.

An array of 21 VR-2 receivers (Vemco Ltd., Nova Scotia, Canada) were anchored to the substrate in various locations at GRMR from May 2004 to May 2008 (Figure 1) to monitor shark presence or absence. Fifteen receivers were arranged in a roughly elliptical transect along the edge of the reef slope surrounding the entire atoll, at depths of 15–30 m. Receivers were attached with shackles and heavy duty plastic cable-ties to a length of polyurethane braided rope, anchored to the substrate by cement blocks chained together, and held upright in the water column by a subsurface float. The remaining receivers were positioned inside the atoll using a similar anchoring system at depths of 2.5–19 m. The position of each receiver was obtained using a hand-held Garmin GPS and plotted on an ArcGIS generated map of GRMR. Field testing indicated that the maximum detection range for these receivers was approximately 300 m (V9 transmitters) and 500 m (V16 transmitters) for units on the reef, and 200 m (V9) and 300 m (V16) for those inside the lagoon. Receivers were collected by SCUBA divers each May and October, their data downloaded and the units refurbished and returned to GRMR. Minor variations in the array configuration occurred between monitoring sessions due to occasional receiver malfunction and theft. We estimated total array coverage was ∼6% of the reef platform. The array was not expected to provide continuous monitoring of shark movements, but rather to detect whether sharks were present at GRMR on any given day.

### Acoustic monitoring analysis

Detections from all receivers were sorted by transmitter, date and receiver to generate a complete monitoring record for each individual implanted with an acoustic tag. We only used strings of two or more consecutive detections for downstream analysis to avoid using spurious detections that arise from signal collisions or background noise. Caribbean reef shark movements were visualized by plotting presence/absence data, gathered from receivers in the array, over a map of GRMR. Three metrics of shark presence and movement within the array were calculated to test the hypothesis that Caribbean reef sharks exhibit a high degree of site-fidelity to GRMR. The distance between the sharks original capture location and that of each receiver at which it was detected was measured using ArcGIS. This was used to calculate “minimum linear dispersal” (MLD) for each individual, defined as the distance between the two furthest receivers at which it was ever detected. “Monitoring duration” was defined as the number of days elapsed between the date of tagging and the date of the last detection string. Because there is some variation in how long transmitters continue to produce detectable signals after their battery expiry date, a standardized “residency index” (RI) was also calculated for all sharks. RI was defined as the total number of days the shark was detected within the array divided by the number of days it could possibly be detected assuming its transmitter worked only up until the expiry date. Any detections recorded for an individual shark that occurred after the estimated battery life expired were not used to calculate RI. Linear regression was used to test for the effect of increasing shark size (age) on RI and MLD.

If sharks exhibit fine-scale site-fidelity to certain parts of GRMR, then the number of detections on a monitor should decrease with distance from the shark's tagging location. The fraction of days each shark was detected by each monitor was modeled using a delta-lognormal approach [27], in which the probability of each reef shark being detected on at least one day during the study was modeled using a logit-link generalized linear mixed model (GLMM) appropriate for binomial (presence/absence) data [28] and the fraction of days observed if present was modeled as lognormal. Potential explanatory variables were: (1) the log of the distance from the shark's tagging location to the receiver; (2) the habitat type at the receiver (ocean reef [n = 15], deep lagoon [n = 3], or shallow lagoon [n = 3]); (3) the individual receivers as random effects; (4) shark type (adult female, adult male, juvenile female or juvenile male); (5) the individual sharks (n = 33, [one shark was never detected]) as a random effect; (6) the number of days the receiver was operational while the shark was tagged (a numerical variable with values 180, 360 and 540, used only for the presence/absence model); and (7) the interactions between ldist and habitat, monitor, shark or shark type. Explanatory variables were included in the model if they were significant, explained more than 2% of the variance, and improved either the Bayesian Information Criterion (BIC) or the Akaike's Information Criterion (AIC) of the model. Analyses were conducted in R, using the MASS and lme4 libraries [28]–[30]. The best model was used to predict whether each shark would be detected at each receiver by rounding the expected probability of detection to zero or one. The expected fraction of days with a detection for each shark×receiver combination was calculated as the probability of any detection from the binomial model multiplied by the expected fraction of days with a detection from the log normal model [27].

A logistic (logit-link) generalized linear model (GLM) was used to predict the presence or absence of each shark anywhere in the receiver array by calendar month. The potential explanatory variables were: (1) the month, counted from when the shark was tagged, as a numerical variable and (2) the transmitter type (12 month versus 18 month battery life). The AIC was used to find the best model. Although some sharks were detected after the end of the assumed battery life of their transmitter, only data from within the first 12 months for each individual was included in this analysis so that all sharks could be compared. The two externally tagged individuals (3291, 3292) were omitted from this analysis in order to maintain continuity.

### Baited remote underwater video (BRUV)

**Figure 2:**
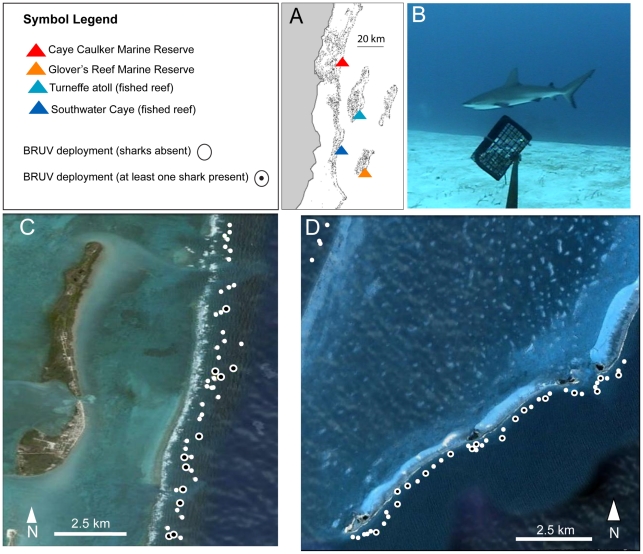
Deployment locations for Baited Remote Underwater Video surveys. (A) Location of the four study sites along the Belizean coast and Mesoamerican barrier reef: Caye Caulker Marine Reserve (CCMR), Turneffe atoll (TU), Southwater Caye (SWC) and Glover's Reef Marine Reserve (GRMR). (B) Still image captured from a BRUV deployment at GRMR with a Caribbean reef shark in frame. (C) Position of BRUV deployments (see symbol legend) at GRMR. (D). Position of BRUV deployments at CCMR.

Caribbean reef shark abundance was surveyed on the fore-reef at four sites (GRMR, [Figure 2] and three other sites, see next section) using baited remote underwater video (BRUV). BRUVs consist of a video camera (Sony Handycam DCR-HC52) inside an underwater housing that is mounted on a metal frame with a small, pre-weighed bait source (1 kg of crushed baitfish) mounted on a pole in the camera's field of view (see [31] for more detail on BRUV design). Data from studies using BRUVs have previously been found to compare well with that obtained from underwater visual census techniques and from baited hook and lines methods for sampling relatively common species [16], [31]–[39]. BRUV sampling locations were chosen for each site by using a random number generator to produce latitude and longitude points on the fore-reef of each site from a map constructed using ArcGIS software. BRUVs were then deployed in these randomly selected locations during daylight hours. Upon arrival at a sampling location, the vessel captain would find the closest suitable location for deployment (an area at a depth of 10–25 m and with bottom substrate flat enough to maximize line of sight). The BRUV was deployed from the boat using a rope and in-water personnel to guide it away from live coral and to orient the BRUV facing down current. The BRUV was left for at least 90 minutes, allowing it to film continuously for ∼85 min after settling to the bottom. No BRUVs were simultaneously deployed within 1 km of another. Units were manually retrieved using the rope, which terminated in a small marker float to facilitate relocation. At both the start and end of each deployment environmental variables were measured including mid water current speed and direction (with a General Oceanics, Mechanical Flowmeter), bottom depth (Lowrance XD85), underwater visibility (secchi disc) and water temperature, salinity, pH and dissolved oxygen (YSI, R85-25). Post deployment, mini-DV cassettes were rendered to digital format and then viewed at normal play speed by one experienced observer (MB). Putative Caribbean reef shark observations were time-logged and then species identity was verified by a second experienced observer (DC). There are no other common carcharhinids likely to be mistaken for this species in the study area [21]. All BRUV deployments were scored as “1” or “0” corresponding to Caribbean reef sharks being “present” or “absent” respectively. Additionally two estimates of the maximum number of Caribbean reef sharks observed per deployment were made: the maximum number Caribbean reef sharks observed in a single frame (Nmax) and the maximum number of individuals observed based on visually definitive differences in body size, sex or markings (Nmax-A).

### Additional BRUV survey sites

**Figure 3:**
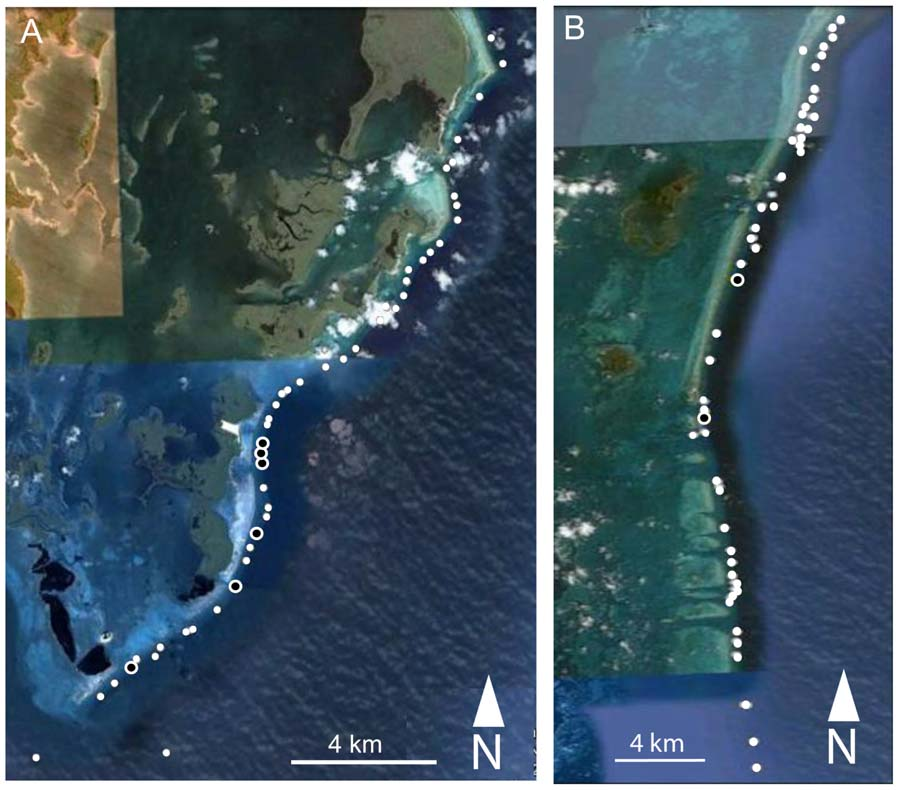
The satellite images show the location of the BRUV deployments (see symbol legend, figure 2) made at the two fished sites: A. Turneffe atoll (TU). B. Southwater Caye (SWC).

BRUVs were also deployed at two fished sites and one additional reserve site in order to compare relative abundance between these reefs and GRMR (Figures 2 and 3). Caye Caulker (17°44′N, 88°1′W) lies 1.8 km to the west of the Mesoamerican Barrier Reef and approximately 20 km to the east of the Belizean mainland. It is a sandbar approximately 7.5 km in length and 1.1 km wide, lying over a limestone shelf. The Caye Caulker Marine Reserve (CCMR) was established in 1998 and is co-managed by the Forest and Marine Reserve Association of Caye Caulker (FAMRACC) and the Government of Belize Department of Fisheries. The CCMR is 1,545 hectares in size, extending 1.6 km beyond the barrier reef. A community-based management program works in concert with the Fisheries Department rangers that conduct all day patrols of the reserve. BRUVs were deployed at CCMR in the same way described previously for GRMR, along a ∼10 km stretch of the fore-reef contained within the marine reserve. Turneffe Atoll (“TU”; 17°21′N, 87°51′W) lies approximately 12 km to the east of the Mesoamerican Barrier Reef and 43 km from mainland Belize. TU is approximately 42 km long and has a maximum width of 14 km. It includes 11 sandy cayes fringed by mangroves arranged around a central lagoon. The majority of the cayes are unpopulated however a few larger cayes, namely Blackbird and Laughing Bird Caye, accommodate dive-based and recreational fishing-based tourism resorts. TU is unique, as it is the only one of Belize's three atolls which is completely open to commercial fishing. BRUVs were deployed at TU in the same way described for the other sites, along ∼23 km of the fore-reef on the southeast of the atoll. Southwater Caye (16°48′N, 88°04′W; SWC) lies on the Mesoamerican Barrier Reef, approximately 19 km to the east of mainland Belize. SWC is a sand island approximately 610 m long and a maximum of 200 m wide, which accommodates two small tourist resorts and a research station. Given its close proximity to the mainland and more densely populated islands it has been subjected to heavy exploitation from commercial fisherman. In 2010 SWC became a marine reserve and active enforcement of the reserve by Fisheries officers began in early 2011. Because enforcement was initiated after we completed sampling, we consider it a fished site for this study. BRUVs were deployed in the same way described for the other sites, across ∼28 km of the fore-reef. Caribbean reef sharks are present in all 4 study sites and are exploited at TU and SWC by fishermen, who deploy large monofilament gillnets and longlines to target sharks for their fins and meat (D. Chapman unpubl. data).

### BRUV analysis

Analysis of presence and absence data was performed by fitting a logitlink GLM. The R software was used with the MASS4 library [38], [39]. The GLM was used to examine the effects of reserve versus non-reserve, location nested within reserve or non-reserve, flow velocity and water temperature on reef shark abundance.

## Results

### Acoustic monitoring at GRMR

A total of 34 Caribbean reef sharks were captured and fitted with transmitters (32 internal, 2 external; Table 1) of which 21 were females and 13 were males. Individuals ranged in length from 66 to 214 cm (mean = 134.1 cm, std.dev. = 39 cm). Two (9.5%) of the females were judged to be sexually mature on the basis of their size, according to published sizes at maturity for this species [24]. Six males (47%) were judged to be sexually mature based on the presence of large, calcified claspers that freely rotated at the base. Sharks were captured throughout GRMR but the most productive fishing and tagging areas were the reef and lagoon area of the southern entrance to the atoll (receivers 1–4, Figure 1; 15 sharks tagged), the fore-reef and reef slope outside Middle Caye (receivers 6 and 25; 8 sharks tagged) and the fore-reef and reef slope outside Middle and Northeast Cayes (“The Elbow”, receivers 10, 21, 22, 23; 7 sharks tagged). The remaining sharks were captured in other parts of the lagoon (Figure 1). Shark capture data (date of capture, sex, size, transmitter type and subsequent monitoring data) are shown in Table 1. There were 14 sharks tagged with smaller V9 tags (estimated 365 day battery life), while 20 sharks were tagged with larger V16 tags (estimated 540 day battery life). We purposefully put the larger tags in larger sharks because of concerns that small sharks might be adversely affected by the V16 transmitters. As a result, the 14 V9 tagged sharks were smaller than the 20 V16 tagged sharks (Table 1).

**Table 1:** Caribbean reef sharks tagged with acoustic transmitters at GRMR.

ID	T Date	Sex	TL (cm)	Location	N days	DUR (days)	MLD (km)	RI
**18**	5/3/2007	F	110	MC	259	359	17.92	0.71
**19**	5/9/2007	F	119	MC	361	356	5.44	0.99
**20**	5/1/2007	M	96	EL	111	362	5.44	0.30
**21**	5/2/2007	M	119	EL	320	360	17.92	0.88
**22**	5/3/2007	F	66	MC	84	229	13.74	0.23
**23**	5/16/2007	F	91	EL	270	348	5.44	0.74
**223**	5/3/2007	F	90	MC	229	359	17.92	0.63
**234**	5/9/2006	F	80	SE	348	355	9.15	0.95
**235**	5/25/2006	F	90	EL	204	321	1.25	0.56
**236**	5/12/2006	M	135	SE	134	426	17.92	0.37
**237**	5/25/2006	F	85	MC	43	468	4.33	0.12
**238**	5/3/2006	M	101	SE	198	362	8.44	0.54
**239**	5/6/2006	M	86	EL	161	475	1.25	0.44
**240**	5/1/2006	F	120	SE	17	147	5.8	0.05
3291*	5/1/2006	F	214	LAG	24	87	5.56	0.04
3292*	10/10/2006	F	214	EL	215	220	11.56	0.39
3346	8/15/2006	F	135	SE	86	274	24.21	0.16
3348	5/5/2004	M	188	MC	386	484	36.46	0.71
3349	5/12/2004	F	134	SE	76	327	36.36	0.14
3372	5/12/2006	F	136	SE	69	358	7.61	0.13
3373	7/2/2005	F	110	SE	170	424	36.46	0.31
3374	5/28/2005	F	142	SE	50	175	3.14	0.09
3376	10/13/2006	F	176	SE	146	303	8.53	0.27
3378	5/11/2007	F	156	NLAG	17	65	26.59	0.05
3379	5/9/2006	M	166	SE	136	466	13.11	0.25
3383	5/31/2005	F	124	SE	143	510	36.46	0.26
3391	5/24/2005	M	167	EL	534	585	20.81	0.98
3340	5/6/2004	M	197	SE	403	534	28.5	0.74
3393	5/6/2004	M	117	SE	458	506	28.5	0.84
4603	12/18/2007	M	176	MC	47	134	4.66	0.31
4604	5/21/2007	F	122	WLAG	5	44	1.25	0.01
4607	5/7/2007	M	151	MC	189	184	23.92	0.52
4608	5/7/2007	M	183	SE	236	359	13.58	0.65

ID = transmitter identity (bolded are V9 tags); T Date = tagging date; TL = total length; Location = Tagging location (see Figure 1); N Days = total number of days with a detection anywhere within the array; DUR = duration between date of tagging and last day detected; MLD = minimum liner dispersal or distance between two furthest receivers with detections; RI = residency index.

* = shark tagged with external rather than internal transmitter.

**Figure 4:**
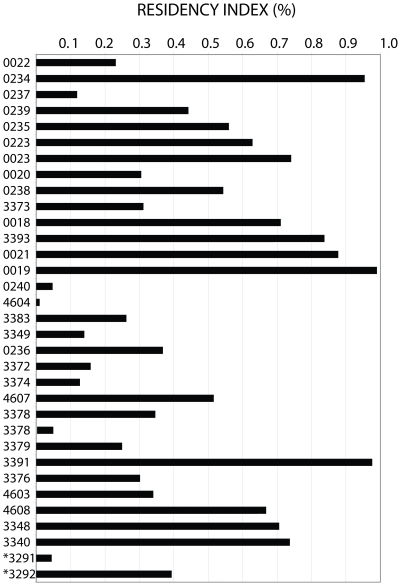
Residency index (RI) of Caribbean reef sharks tagged at GRMR with acoustic transmitters. Individual sharks are denoted by their transmitter code (see Table 1) and are arranged by increasing body size from top to bottom. (*) indicates the shark was fitted with an external transmitter as opposed to having one implanted into its coelom.

**Figure 5:**
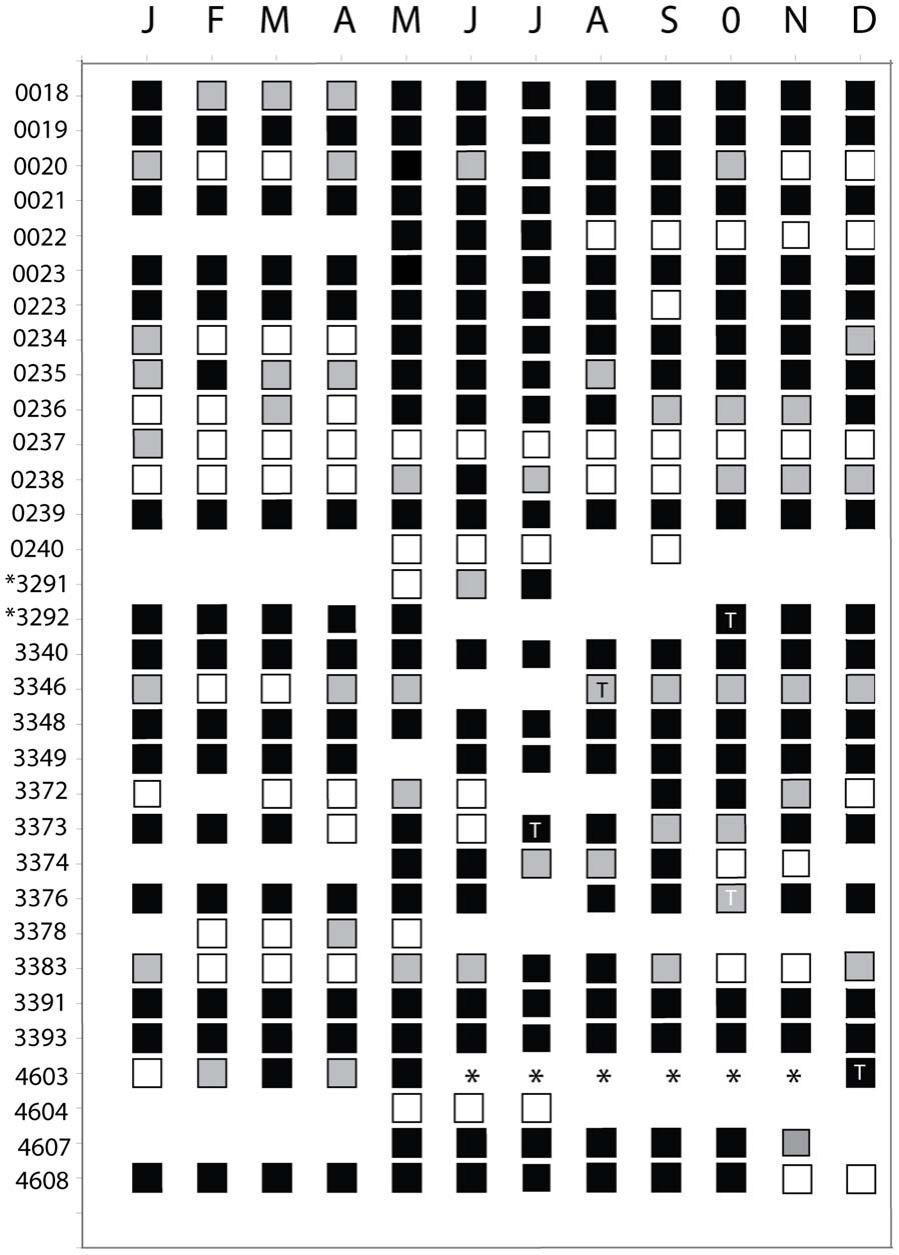
Monthly occurrence of each of the tagged sharks at GRMR. A square denotes that the individual (transmitter code on y-axis) was detected on at least one day during the given month. The color of the squares indicates the number of days that month that the individual was detected with the scale of white (1–7 days), grey (8–14 days) and black (>14 days). The values are total number of days per month not consecutive days. “T” denotes the tagging time of sharks not tagged in the month of May or June and “*” refers to a break in receiver coverage due to refurbishment or damage.

All but one of the tagged Caribbean reef sharks were detected after release. Single, isolated detections were excluded from the analysis due to the possibility of them being spurious detections. It was highly unusual for more than two individuals to be detected simultaneously on the same receiver. When three individuals were detected simultaneously at a receiver we attempted to verify that detections of the third individual were not an artifact of signal collisions between the other two, which we reasoned would consist of detection strings with an unusually long lag time between detections given the transmission rate of the transmitter. No detection strings met this criterion. Both the total number of days that each individual was detected and the monitoring duration within the array was related to the tag type. For sharks tagged with V9 transmitters (N = 14), the number of days detected ranged from 17–361 days (mean = 195 days, std. dev. = 109 days; Table 1) and the monitoring duration ranged from 147–468 days (mean = 351 days, std. dev. = 84 days; Table 1). For sharks tagged with V16 transmitters and tracked until the battery life expired (N = 14; 5 V16-tagged sharks were tracked for ∼65% of potential tag battery life because they were tagged late in the study), the number of days detected ranged from 5 to 534 days (mean = 178 days, std. dev. = 163 days; Table 1) and the monitoring duration ranged from 65–585 days (mean = 382.1 days, std. dev. = 171 days; Table 1). Mean Residency Index (RI) among Caribbean reef sharks was 0.43 (i.e., the “average” shark was detected on 43% of the days it had a functional transmitter and there were receivers in the water, std. dev. = 0.3) and ranged from 0.01 to 0.99. (0.53 and 0.36 were the means for V9 and V16 transmitters respectively; Table 1, Figure 4). RI was higher among sharks tagged off on the fore-reef off Middle Caye and the Elbow (mean RI = 0.53 and 0.65 respectively) compared to sharks tagged in the southern entrance and lagoon (mean RI = 0.33). Most sharks (20, 64%) were detected on at least one day during every month of the year and all but three were detected for six months or more (Figure 5). Individual Minimum Linear Dispersal (MLD) ranged from 1.25–36.4 km (mean 9.4 km, std. dev. 6.3 km for V9; mean 19.3 km, std. dev. 12.6 km for V16, Table 1). Neither RI nor MLD was significantly correlated with shark body size (r^2^ = 0.02 and 0.04 for RI and MLD respectively).

**Figure 6:**
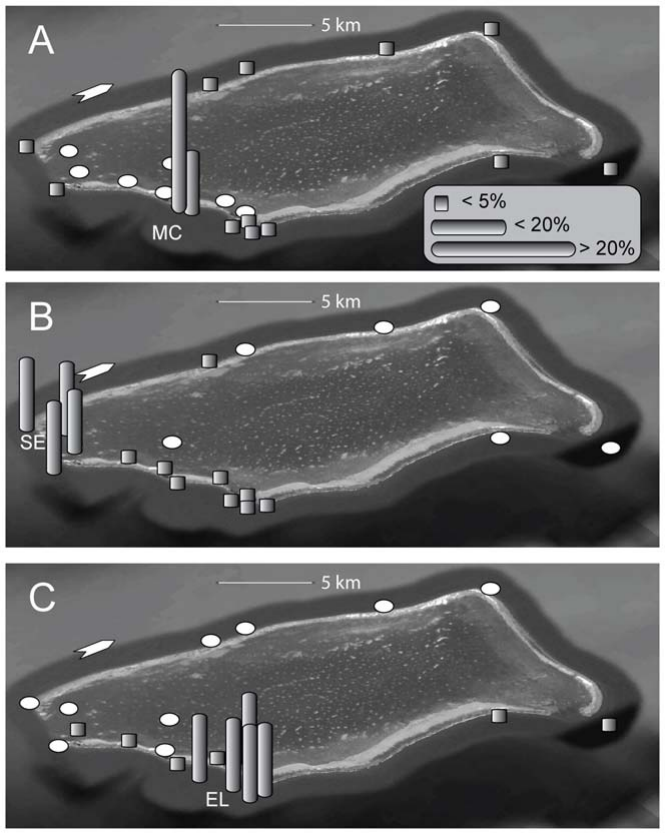
Examples of fidelity of Caribbean reef sharks to sites monitored by receivers. The number of days that all sharks tagged in the specified location were detected anywhere in the array were pooled and then apportioned to receivers. A-Pooled detection days of sharks tagged at Middle Cay (MC); B-Pooled detection days from sharks tagged at Southern Entrance (SE) and C- Pooled detection days of sharks tagged at the Elbow (EL). The height of the bar over each receiver designates the percentage of the pooled days with detections that occurred on that receiver. North is indicated by the arrow. Flat circles show receivers with no detections for any sharks tagged in the specific location.

**Figure 7:**
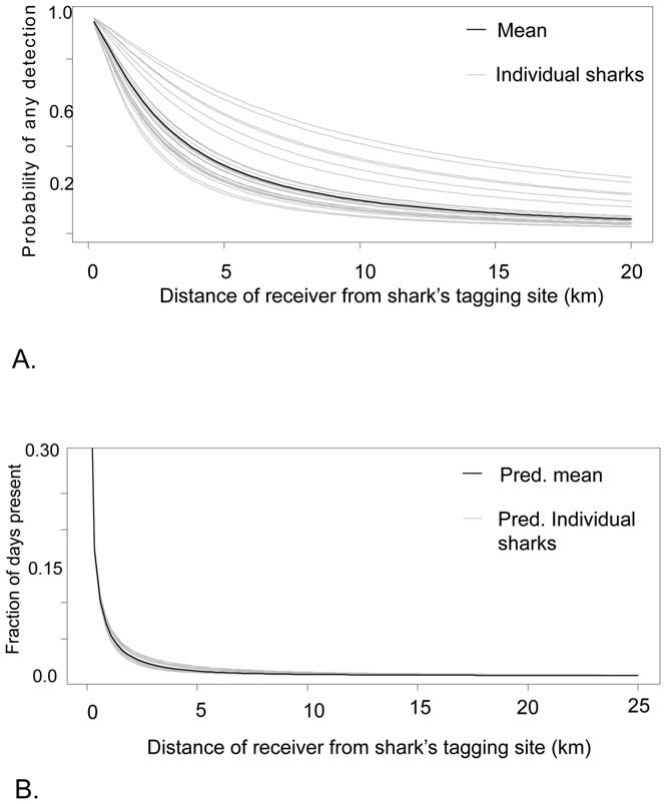
The influence of distance of receiver from sharks tagging site on the probability of detection and number of days detected. (a) Probability of detection from the AIC best model of presence/absence, for a receiver operational for one year, in the ocean reef habitat (b) Lognormal predicted fraction of days observed from the two models combined.

All Caribbean reef sharks were primarily detected on one or a small number of receivers (Figure 6). The most important factor influencing whether a Caribbean reef shark was ever recorded at a receiver was the log distance between the receiver and the shark's original tagging location; this factor alone explained 23% of the deviance in the presence/absence data (Table 2). There were also significant effects of the number of days sampled and habitat, as well as significant variation between individual sharks. The best mixed effects model according to the AIC (Table 3) allowed the effect of shark length to vary by individual shark. The AIC best fit model correctly predicted the presence or absence of individual reef sharks 83% of the time (Table 4). A model in which days sampled and log distance were the only explanatory variables correctly predicted individual reef shark presence or absence 81% of the time (Table 5). The fraction of days with a detection (given any detection) declined significantly with distance (Tables 6, 7 and Figure 7). The AIC best fit model included only log distance and habitat, and their interaction. Because the log-distance habitat interaction was not significant we excluded it from further consideration. Log distance explained 36% of the total deviance. Because the expected probability of detection varied by shark type and other factors, there was considerable variability in the expected number of days observed ( = expected probability of detection times expected number of days detected divided by the number of days sampled, Figure 7). Nevertheless, for all combinations of the explanatory variables, the fraction of days with detections from individual sharks was expected to be less than 10% for distances from the original tagging site greater than 1 km.

**Table 2:** Analysis of deviance for the AIC best model of presence or absence of Caribbean reef sharks by receiver with fixed effects only.

	Df	Deviance	Resid. Df	Resid. Dev	P(Chi)	Percent deviance
NULL			591	700.62		
days	1	18.06	590	682.55	0.00	0.03
ldist	1	158.43	589	524.13	0.00	0.23
shark type	3	6.13	586	517.99	0.11	0.01
habitat	2	7.05	584	510.94	0.03	0.01

“Days” refers to days sampled by each receiver; “ldist” is log(distance).

**Table 3:** The AIC and BIC values for models with random effects (in bold).

Model	AIC	BIC	deviance	delta.BIC	delta.AIC
days+ldist+habitat	526.71	548.63	516.71	14.43	17.65
days+ldist+habitat+shark	513.56	539.86	501.56	5.65	4.49
days+ldist+monitor	529.87	547.41	521.87	13.20	20.81
days+ldist+monitor+shark	515.61	537.52	505.61	3.32	6.54
days+ldist+habitat+ldist×shark*	**509.06**	**544.13**	493.06	9.93	0.00
days+ldist+ldist×monitor	**530.77**	**557.07**	518.77	22.86	21.70
days+ldist+ldist×monitor+ldist×shark	**513.96**	**553.42**	495.96	19.21	4.90
days+ldist	529.62	542.77	523.62	8.56	20.55
days+ldist+shark	516.67	534.20	508.67	0.00	7.61
days+ldist+ldist×shark	**519.46**	**545.76**	507.46	11.56	10.40

**Table 4:** Observed and predicted presence or absence of Caribbean reef sharks based on the AIC best model (days+ldist+habitat+ldist×shark).

	Observed	
Predicted	absent	present
absent	416	92
present	11	73

**Table 5:** Observed and predicted presence or absence of Caribbean reef sharks based on a model including only log-distance and days sampled.

	Observed	
Predicted	absent	present
absent	403	90
present	24	75

**Table 6:** AIC best model of log of days with a detection for the fraction of days each shark was observed at each monitor.

	Df	Deviance	Resid. Df	Resid. Dev	F	Pr(>F)	Percent deviance
NULL			164	582.06			
ldist	1	211.33	163	370.73	101.87	0	0.36
habitat	2	28.31	161	342.42	6.82	0.001	0.05
ldist∶habitat	2	12.57	159	329.85	3.03	0.051	0.02

**Table 7:** AIC best model of log of days with a detection for models with additional random effects.

Model	AIC	BIC	deviance	delta.BIC	delta.AIC
ldist	607.82	617.14	370.73	2.89	11.28
ldist+habitat	598.72	614.25	342.42	0.00	2.17
ldist+habitat+ldist∶habitat	596.54	618.29	329.85	4.04	0.00
ldist+habitat+ldist∶habitat+**shark**	603.43	628.28	582.54	14.03	6.89
ldist+habitat+ldist∶habitat+**monitor**	602.61	627.46	582.73	13.21	6.06
ldist+habitat+ldist∶habitat+**monitor**+**shark**	604.61	632.56	582.73	18.32	8.06
ldist+habitat+ldist∶habitat+**ldist×monitor**	602.50	627.35	582.48	13.11	5.96
ldist+habitat+ldist∶habitat+**ldist×monitor**+**ldist×shark**	604.42	632.37	582.54	18.12	7.87

**Figure 8:**
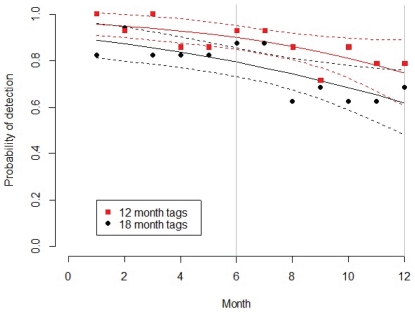
Predicted probability of presence/absence for the AIC best model (solid lines) plus and minus 2 standard errors (dashed lines). Points are the average values in each month in the data for sharks tagged with V9 (12 month) transmitters and V16 (18 month) transmitters, see symbol legend. The y-axis is the predicted presence or absence of the shark with 0 being absent and 1 being present, the x-axis is the duration since the shark was tagged in months.

The predicted probability of presence (detection) of sharks by calendar month from the AIC best model, showed that the probability of a shark being detected remains quite high throughout the first 12 months (Figure 8). Although calendar month and tag type (12 month versus 18 month battery life) both had a significant influence over the probability of presence they explained little of the variance (Table 8). The logistic regression predicts that 70–80% of sharks are still within the array, one year after tagging.

**Table 8:** The AIC best model for the probability of presence, for the duration since tagged (month) and the battery life of the tag.

	Df	Deviance	Resid. Df	Resid. Dev	Pr(>Chi)	Deviance
NULL			364	345.03		
sharkmonth	1	14.008	363	331.02	0.000182	0.04
taglife	1	7.226	362	323.79	0.007185	0.02
—						0.06

### Baited remote underwater video at 4 sites

**Figure 9:**
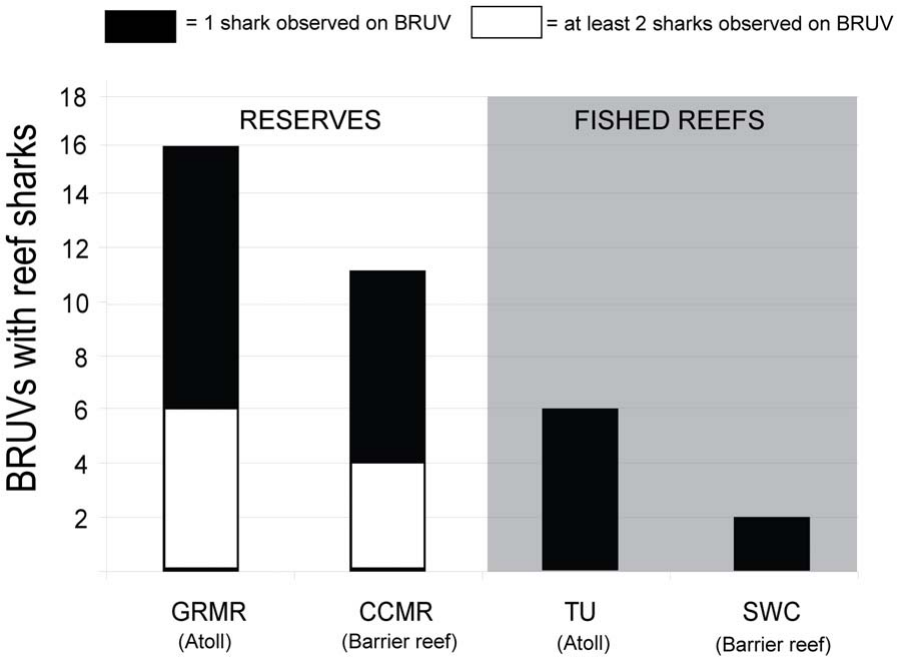
Number of BRUV deployments out of 50 per site in which one (solid portion of bars) or more (open portion of bars) Caribbean reef sharks were recorded at GRMR (reserve), CCAR (reserve), TU (fished) and SWC (fished).

A total of 200 BRUV deployments were made, divided equally across the 4 study sites (total duration of 17,200 minutes). The deployments were made between June 11–19 2009 (BRUV's, n = 44) and May 6–12 2010 (n = 6) at GRMR, with Caribbean reef sharks observed on 16 BRUVs (32% of deployments at this site), 6 of which recorded 2 individuals. At TU deployments occurred between June 21–26 2009 (n = 50), with a shark observed on 6 BRUVs (12% of deployments). Deployments occurred at SWC between July 3–8 2009 (BRUV's n = 39) and May 23–29 2010 (n = 11), with a shark observed on 2 BRUVs, (4% of deployments). CCMR deployments occurred between 30 June–2 July 2009 (BRUVs, n = 21) and 18–20 May 2010 (BRUVs, n = 29) with Caribbean reef sharks being recorded on 13 BRUVs (26% of all deployments), with 6 of these recording 2 individuals in frame at once (3 in 2009 and 3 in 2010). Overall, 35 of the 200 BRUV deployments (17.5% of total number of deployments) recorded at least one Caribbean reef shark. All but 8 of these were in marine reserves, with at least 10 of the marine reserve deployments recording at least 2 different individuals (Figure 9). As a result, whether or not the BRUV was deployed within a marine reserve had a significant impact on reef shark presence in the GLM (Table 9). There was no difference between the two reserve sites or between the two non-reserve sites in reef shark presence/absence.

**Table 9:** The GLM analysis on the influence of the conservation boundaries (reserve and non-reserve) and location, on reef shark presence or absence derived from BRUV deployments.

	Df	Deviance	Resid. Df	Resid. Dev	P(>|Chi|)	percent.deviance
NULL			119	108.135		
Reserve	1	9.063	117	98.636	0.002	0.085
Reserve∶Location	2	2.7504	115	95.885	0.253	0.023

The two reserve sites (GRMR and CCMR) had smaller total areas when compared to that of the two fished sites (TU and SWC). To ensure that the observed increase in relative abundance of reef sharks at the reserve sites was not biased by a greater proportion of the available habitat being sampled, the number of samples was proportionately reduced by bootstrapping according to total area, to attain an equal sample density per site. The reduced numbers of BRUVs per site of TU (n = 50), SWC (n = 30), GRMR (n = 25) and CCMR (n = 25) were randomly subsampled from the complete data set, using the R software [29].The application of GLM to 2000 bootstrapped subsamples found the marine reserve factor to still be the significant (p<0.05) influence on the presence or absence of reef sharks in 84% of the samples. Therefore the difference in size between the reserve and fished sites caused no bias as to the significance of the marine reserve factor and its influence on reef shark relative abundance.

There were also no significant differences in flow, salinity, depth or visibility between locations (ANOVA, P>0.05). Temperature and DO were both significantly higher at SWC than at any of the other three locations (Tukey HSD, p<0.001). Because the vast majority of BRUV deployments in the non-reserve sites resulted in zero reef sharks being observed it was not possible to evaluate the impact of environmental variables and reserve versus non-reserve effects in the same model. For the subset of data from marine reserves, there were no significant effects of site, location, water temperature or flow velocity (Table 10). However, there was a significant interaction between flow velocity and location.

**Table 10:** The GLM analysis on the influence of the environmental parameters (flow velocity and water temperature) on reef shark presence or absence derived from BRUV deployments within the marine reserve sites.

	Df	Deviance	Resid. Df	Resid. Dev	P(>|Chi|)	percent.deviance
NULL			59	69.590		
Location	1	1.375	58	68.215	0.241	0.020
flow	1	2.059	57	66.156	0.151	0.030
start.temp	1	0.149	56	66.007	0.700	0.002
Location∶flow	1	7.955	55	58.053	0.005	0.114
Location∶start.temp	1	0.664	54	57.389	0.415	0.010
flow∶start.temp	1	1.105	53	56.283	0.293	0.016

## Discussion

We tested the hypothesis that Caribbean reef sharks are to benefit from the local respite from fishing occurring within marine reserves by examining two of its key predictions. The first prediction is that Caribbean reef sharks exhibit high site-fidelity to reserve areas. Acoustic monitoring showed that most individuals exhibit a high degree of site-fidelity at GRMR. The mean residency index (RI) indicated that the average shark was detected nearly one out of every two days at GRMR. Notably, RI is a conservative metric considering that receiver array coverage was modest (∼6% of the reef platform). We found that sharks tagged on the fore reef typically had higher RI and were less likely to be lost from the array than lagoon-tagged sharks. These observations most likely reflect differences in receiver coverage and effectiveness between these two reef habitats. Not only were more receivers deployed on the fore-reef, this habitat is so narrow, usually <500 m, that a line of receivers deployed along the reef slope is likely to regularly detect passing sharks. Reef sharks in the lagoon can swim in most directions and may not necessarily swim close to an isolated receiver despite being close to it. Transmitters are also more likely to be detected in the fore-reef because seafloor relief is low relative to water depth, whereas receivers in the lagoon are partially blocked by emergent patch reefs. Notwithstanding the limitations of acoustic monitoring in the lagoon we found that the probability of detecting tagged sharks by calendar month was high throughout the year following transmitter application. This indicates that sharks were typically year-round residents of GRMR as opposed to being seasonal immigrants. Several large individuals tagged with 18 month (i.e., V16) transmitters were also generally detected right up until or slightly beyond projected transmitter battery life, indicating use of the atoll across successive years. Despite reasonably high RI for many individuals it is important to highlight that these sharks are capable of long range movements over short time periods (days [23]). Many individual sharks were sporadically absent from the receiver array, which leaves open the possibility that most sharks occasionally depart GRMR for short periods and may be exposed to fisheries during these movements.

Individual Caribbean reef sharks were mainly detected on a localized subset of receivers within the GRMR array. GLMM analysis indicated that a receiver's distance from the shark's original capture location was an important factor in determining the probability of shark detection. It therefore appears that we typically captured Caribbean reef sharks in an area that they regularly used after release, suggesting that they are a home ranging species (i.e., they regularly use a fraction of the available habitat, rather than moving throughout [40]). Home ranging behavior has been suggested for several tropical carcharhinid sharks, especially species that live on coral reefs, including Caribbean reef sharks in Brazil [14], [19], [41]–[44].

Sharks often exhibit an ontogenetic expansion of home range size [45] and we would expect a positive correlation between MLD and shark size if this is true for Caribbean reef sharks. Moreover, we would predict a negative correlation between RI and size if large sharks leave GRMR more than small ones. None of the metrics we were able to calculate from monitoring data, however, demonstrated a significant correlation with body size. We suggest that it is still reasonable to hypothesize that large juvenile and adult Caribbean reef sharks have larger home ranges than small juveniles and we recommend that active telemetry tracking should be used to generate activity space metrics (e.g. estimated home range size) that could be more readily compared between individuals than the coarse acoustic monitoring data we collected. This type of information is necessary to make more refined predictions about how different life-stages will respond to different sized marine reserves.

Is the high fidelity of Caribbean reef sharks to GRMR largely driven by the isolation of this reef platform? Large individuals of this species monitored and tracked at GRMR moved across pelagic habitat [22] and dove to depths of at least 352 m [23]. These observations suggest that deep, open water separating GRMR from the barrier reef and other atolls is not an insurmountable barrier to dispersal that forces high site-fidelity. We hypothesize that this species may naturally exhibit high site- fidelity, with the degree of fidelity possibly a function of the reef's isolation, climate and carrying capacity. For example, sharks may move between proximate reefs; they may migrate at higher latitude reefs in response to seasonal temperature changes and they may be more likely to emigrate from a reef as competitor density increases or prey availability decreases. Caribbean reef sharks could be acoustically monitored at reefs of different levels of isolation, latitude and prey abundance to further test these hypotheses.

The second prediction of our main hypothesis is that the relative abundance of Caribbean reef sharks is higher in reserves than similar fished reefs. The factor “marine reserve” was the most important predictor of shark presence or absence on BRUVs in the GLM, which is consistent with this prediction. This analysis assumes that random sampling of each site conducted over a few days is representative of relative abundance throughout the year. We suggest that is a reasonable assumption given the high degree of site-fidelity we observed at GRMR using acoustic monitoring and results from other telemetry studies of this species [14], [15], [26]. Nearly four times as many BRUVs deployed in marine reserves recorded Caribbean reef sharks than on fished reefs. Several of the reserve-deployed BRUVs also recorded 2 individuals, either in frame at once or identified by visually obvious differences in size or markings. Our results are consistent with studies of reef sharks in Brazil and Australia that also show higher relative abundance in reserves [14], [16]–[18].

The issue of a potential bias arising from the difference in the total area of the reserve sites versus the fished sites was addressed by performing bootstrap sampling techniques. The fished sites had a larger total area to be sampled which could have led to pockets of higher shark abundance being under-sampled due to a lower sample density, when compared to the reserve sites. However, by analyzing reduced sample sizes to correct for the difference in sample density the result was found to be the same, with marine reserve being the only significant predictor of reef shark presence.

The data do not support the competing hypothesis that reef shark relative abundance was primarily driven by environmental variation between sites. Most environmental parameters were not significantly different between the sites and none were consistently different between the reserves and non-reserve sites. All BRUV deployments occurred on ocean-facing fore-reef habitat within 1 km of the reef slope, which is typical habitat for the species [21], [22], [24], [46]. Our data indicates that Caribbean reef shark abundance better tracks the level of local fishing pressure than any of the environmental factors we examined.

Our combined telemetry and survey results support the hypothesis that marine reserves can have a positive effect on the local abundance of reef sharks or at least significantly reduce rates of population decline relative to fished ones. Time series of shark abundance inside marine reserves (e.g., [21], which confirms that GRMR had a stable Caribbean reef shark catch per unit effort from 2001–2005) are needed to determine whether high relative abundance also means that populations are stable or increasing. Given growing support for the hypothesis that reef sharks are more abundant inside marine reserves, it is reasonable to speculate about the potential enhancement mechanisms involved. Potential mechanisms include “direct enhancement”, where highly residential reef sharks increase inside marine reserves due to a local respite in fishing pressure on them. A second potential mechanism is “increased prey”, in which reserve areas support larger reef shark populations because reserves provide a local respite in fishing pressure for the sharks' prey. Studies that monitor changes in reef shark populations and their prey communities before and after reserve establishment are needed to study the relative importance of these enhancement mechanisms.

In conclusion, our telemetry and BRUV survey results support the hypothesis that Caribbean reef shark populations can benefit from the local respite from fishing pressure provided by marine reserves. Of course, reserve size, placement and compliance will influence whether or not these benefits materialize. We suggest “direct enhancement” and “increased prey” as potential enhancement mechanisms. Our study also underscores that Caribbean reef shark abundance on some fished parts of the Mesoamerican Barrier Reef is relatively low, which is concerning from the perspectives of fisheries sustainability and ecotourism. Although the ecological role of reef sharks is not well studied, it is possible that the local reduction in these upper level predators has significant effects on the coral reef ecosystem (e.g. [47]). Our study and others show that marine reserves have an increasingly clear role in the conservation of reef sharks. We suggest that reserves-or larger scale area closures- should be considered as an important tool to preserve the ecological and economic roles of reef sharks in increasingly imperiled Caribbean coral reef ecosystems.
